# Persistent psychosis associated with extreme delta brush in anti-NMDA receptor encephalitis: a case report

**DOI:** 10.1186/s12888-023-04750-8

**Published:** 2023-04-17

**Authors:** Paulo Ribeiro Nóbrega, Paulo Reges Oliveira Lima, Pedro Helder de Oliveira Junior¹, Lorena Pitombeira Sanders, Manoel Alves Sobreira-Neto, Samir Câmara Magalhães, Lia Lira Olivier Sanders, Pedro Braga-Neto

**Affiliations:** 1 Division of Neurology, Department of Clinical Medicine, Universidade Federal do Ceará, Ceará, Brazil; 2 Center of Health Sciences, Universidade Estadual do Ceará, Ceará, Brazil; 3 Universidade de Fortaleza, Fortaleza, Brazil; 4 Division of Psychiatry, Department of Clinical Medicine, Universidade Federal do Ceará, Ceará, Brazil

**Keywords:** Autoimmune diseases, Encephalitis, Anti-N-Methyl-D-Aspartate receptor encephalitis, Seizure, Extreme delta brush

## Abstract

**Background:**

Anti-NMDAR encephalitis is an emerging differential diagnosis of first episode and persistent psychosis in the psychiatric community, as clinical manifestations include psychiatric symptoms, cognitive dysfunction, seizures, decreased consciousness, and dyskinesias. This disease is associated with extreme delta brush (EDB), but the significance and temporal course of this EEG pattern still needs to be determined. Herein, we report a case of anti-NMDAR encephalitis with persistent psychosis associated with EDB occurrence on multiple occasions during a 5-year disease course.

**Case presentation:**

A 15-year-old girl was diagnosed with anti-NMDAR encephalitis and treated with progressive improvement. Four years after initial manifestations, an EDB pattern was seen on electroencephalogram (EEG) without new neurological symptoms. She had residual symptoms of episodic auditory hallucinations and impulsivity. One year later, the patient had a recurrence of neurological symptoms (seizures, dyskinesias and impaired attention), persisting with EDB on EEG. Clinical symptoms and EDB resolved after second-line treatment with rituximab.

**Conclusion:**

We describe the first case of persistent psychosis in anti-NMDAR encephalitis associated with extreme delta brush on multiple EEGs on prolonged follow-up. Electroencephalographic patterns such as EDB may serve as markers of residual disease activity, including psychiatric symptoms. Further studies with prolonged EEG monitoring are needed to better understand these findings.

## Background

Anti-N-Methyl-D-Aspartate Receptor (NMDAR) encephalitis is the leading cause of encephalitis in patients under the age of 30 [1]. Patients may experience a flu-like prodrome followed by severe psychiatric symptoms, memory loss, seizures, and decreased consciousness. Sometimes a preceding viral illness may act as a trigger for autoimmunity [2–4]. Adults present with more psychiatric and cognitive disorders than children [5]. Therefore, a significant proportion of patients with anti-NMDA receptor encephalitis may initially seek the help of a psychiatrist [6]. Anti-NMDAR encephalitis is caused by immunoglobulin G (IgG) antibodies against the GluN1 subunit of the NMDAR [7].

Extreme delta brush (EDB) is an electroencephalographic pattern sometimes observed in this disease, although not unique to anti-NMDA-R encephalitis [8]. EDB consists of synchronous and symmetric 1–3 Hz waveforms superimposed by a burst of rhythmic 12-30-Hz activity in frontotemporal regions. This pattern usually disappears after treatment with anti-epileptics but sometimes may persist for several months. EDB is associated with more severe and prolonged forms of the disease [9]. However, it is not clear how EBD relates to persistent disease activity in anti-NMDA-R encephalitis [1].

Herein, we report a case of anti-NMDAR encephalitis with persistent psychotic symptoms associated with EDB occurrence on multiple occasions during a 5-year disease course.

## Case presentation

A 15-year-old female patient was taken by her parents to a psychiatric emergency room presenting auditory and visual hallucinations as well as delusions (described as command hallucinations, thought broadcasting and persecutory delusions.) in March 2015, characterizing a first episode of psychosis. After two months, she had progressive impairment of speech and gait, along with fever, rigidity, and seizures. The patient was impulsive, hypersexualized, and disinhibited. There was no previous history of medical or psychiatric conditions, drug abuse, or family history of neurologic diseases.

**Fig. 1:**
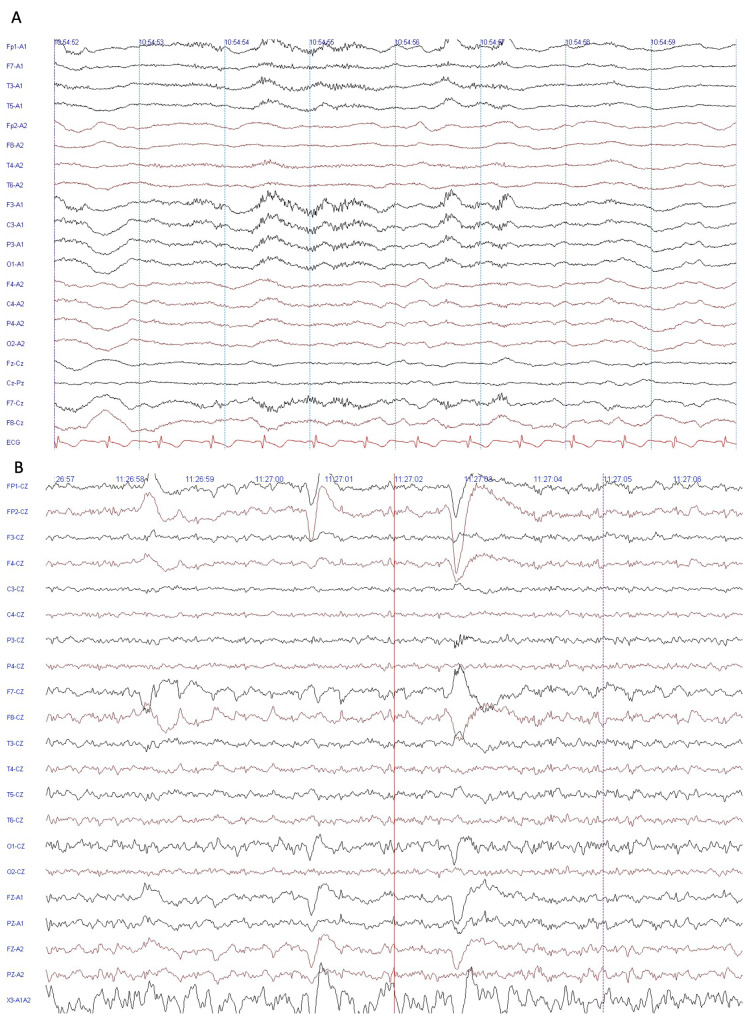
A - EEG during wakefulness showing slowing of background activity and extreme delta brushes (EDBs) predominantly in the left frontal region. B - EEG during somnolence (sleep stage N1) with predominantly irregular delta rhythms without EDB. EEG parameters: sensitivity 15 µV/mm; High frequency filter 70 Hz; low frequency filter 0,5 Hz; notch filter on; sampling rate: 1024 Hz; acquisition speed 30 mm/second

On admission, in June 2015, she was catatonic (she scored 11/14 in the Bush–Francis Catatonia Screening Instrument, which has been previously used to assess catatonia in anti-NMDAR encephalitis) [10], had continuous orofacial and hand dyskinesias, autonomic dysfunction and horizontal nystagmus. Serologic tests were negative for HIV, hepatitis, syphilis, rheumatoid factor, antinuclear antibody, and ANCA. Cerebrospinal fluid (CSF) showed 2 cells/mm^3^ and protein levels of 57 mg/dL Magnetic resonance imaging (MRI) revealed small bilateral areas of encephalomalacia at the posterior occipital lobes. An electroencephalogram (EEG) identified EDB (Fig. 1A) At this point she had frequent motor seizures and was comatose. She received methylprednisolone 1 g/day for five days and immunoglobulin (IVIG) 400 mg/kg/day for five days without any adverse events. Anti-NMDAR immunoglobulin G (IgG) antibody was positive in CSF (titers not recorded). Computed tomography (CT) of the chest, abdomen and pelvis, pelvic MRI, and transvaginal ultrasound were negative for malignancy. Seizures subsided and dyskinesias improved.

After six months of follow-up, there was a significant improvement in cognitive functions (Mini-Mental State Examination [MMSE] 27/30), Auditory hallucinations improved after the use of quetiapine 300 mg/day.

From 2016 to 2019, she remained neurologically stable, with preserved cognitive function and no seizures. The only symptoms that remained were occasional auditory hallucinations and impulsivity. We have no follow-up EEG from this period. In March 2019, a new EEG was requested because of persistent psychiatric symptoms and revealed extreme delta brush (Fig. 1A). We also requested a new CSF anti-NMDAR test, but it was not performed due to social factors. Hallucinations resolved and impulsivity improved with risperidone 2 mg/day. The patient missed the following appointments and therefore no treatment was started.

In August 2020, five years after initial manifestations of anti-NMDAR encephalitis, she presented irritability, apathy, fluctuating attention, social isolation, seizures, impairment of speech, nystagmus and orofacial dyskinesias. We initiated intravenous methylprednisolone (1 g/day for five days) and IVIG (400 mg/kg/day for five days). Electroencephalogram performed in September 2020 demonstrated persistence of EDB. Pelvic MRI and screening for infections were negative. Anti-NMDAR IgG antibody was positive in the serum (titer 1:200) and CSF (titer 1:64).

**Fig. 2:**
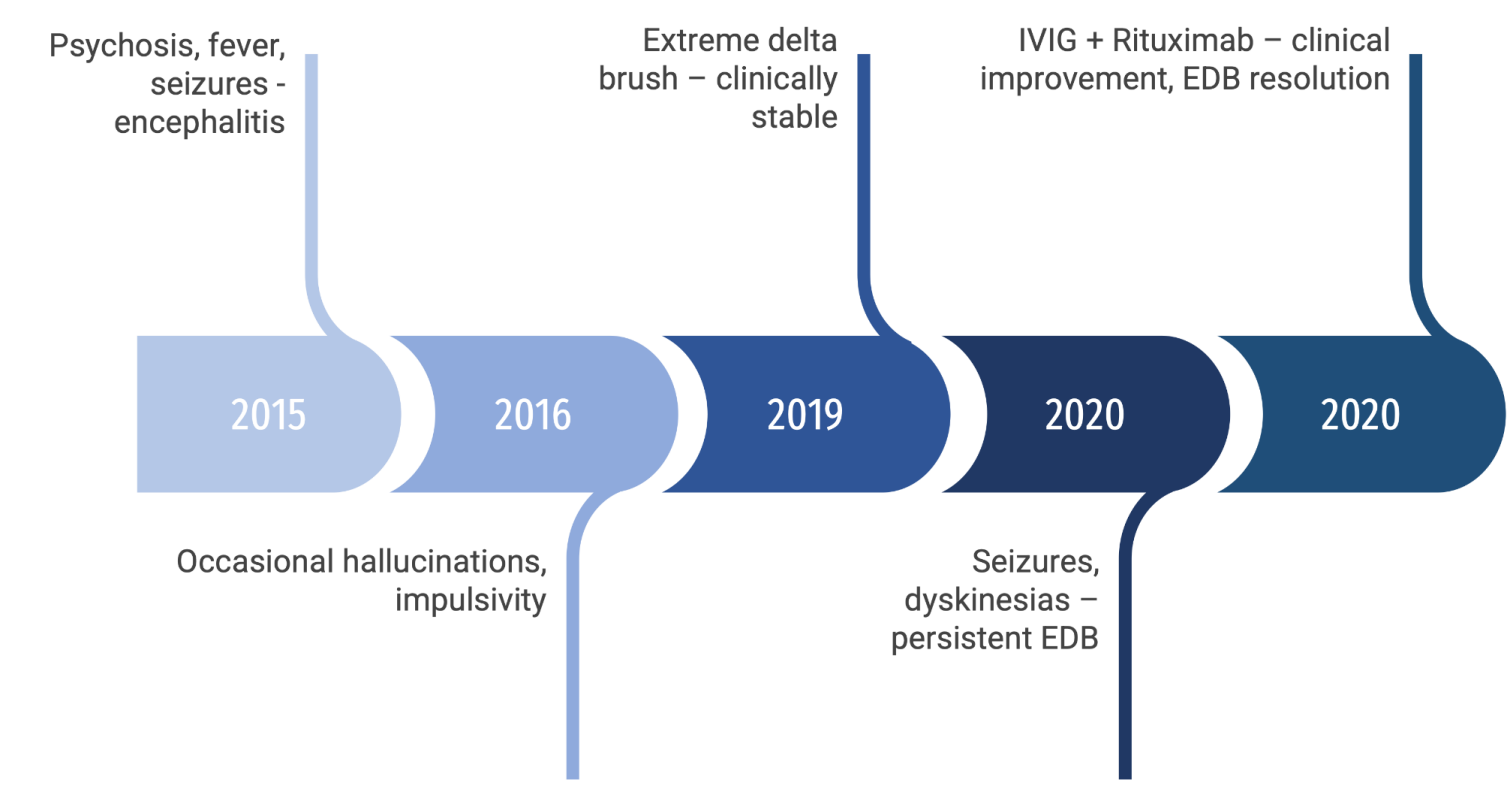
Timeline showing the disease course in a patient with anti-NMDA encephalitis and persistent extreme delta brush

One month after IVIG, she had an improvement in seizures and dyskinesias, but maintained impulsive behavior and auditory hallucinations. We then prescribed Cyclophosphamide 750 mg/m2 and rituximab 750 mg/m2. Dyskinesias resolved, and hallucinations subsided. Her most recent EEG (January 2021) revealed normal baseline activity without EDB (Fig. 1B). Disease course timeline is shown in Fig. 2. The patient is back to her previous functioning in daily activities and her latest MMSE is 27/30.

## Discussion and conclusions

We describe the first case of anti-NMDAR encephalitis with persistent psychosis and EDB. The significance of EDB in the follow-up of this population is unknown, and this finding raises the possibility of EDB as a biomarker for residual disease activity.

Extreme delta brush occurs in around 22% of anti-NMDAR encephalitis patients. It appears after some days of clinical symptoms and may reflect a disruption of the rhythmic neuronal activity in anti-NMDAR encephalitis [11]. A study by Zhang and colleagues showed, in a small number of patients, that EDB disappears six months after therapy in all patients and that the variable prevalence of this finding could be related to the timing of EEG [12]. They did not mention residual symptoms in these patients. Our patient persisted with psychiatric symptoms, partially controlled by neuroleptics, which could indicate residual disease activity.

Psychiatric symptoms, including first-episode psychosis, are common in anti-NMDA encephalitis. A review of published cases in Latin America shows that they occur in up to 67.11% of patients as the most common presenting feature [13]. Nevertheless, a recent review found no cases of anti-NMDAR encephalitis in isolated first-episode psychosis [5]. However, psychiatric symptoms may represent residual disease activity and might be the predominant features during relapses. Irritability, aberrant motor behavior, and sleep behavior disorders are the most common persistent neuropsychiatric manifestations. Hallucinations are observed in only 1.9% of patients after 24 months of follow-up [14].

Around 12–25% of patients might relapse [9, 15]. Among ten patients with anti-NMDA encephalitis followed up at our center for up to 8 years [16], this was the only one to suffer a relapse. Clinical monitoring for relapse is often tricky as symptoms might be milder [9, 15], CSF antibody titers do not correlate accurately with disease activity [9, 17], and MRI findings are not specific in most cases. Relapses are less common in patients treated with second-line therapy with rituximab [18, 19]. This patient received only first-line treatment at the initial presentation due to rapid clinical improvement, which might have increased her risk of relapse. At the time, the patient received only prednisone for nine months after initial treatment and no immunosuppressants. Current recommendations suggest immunosuppression for at least one year with mycophenolate mofetil or azathioprine to prevent relapse [9, 15].

Relapsing anti-NMDAR encephalitis is defined as any new psychiatric or neurologic syndrome that cannot be explained by other causes and that improved after immunotherapy or, less frequently, spontaneously, different studies have described abnormalities in CSF, EEG, and MRI. These are not as frequent as in the first episode but still can help guide the diagnosis [20]. We have found no description of EDB during relapses.

Unfortunately, we have not performed EEG after the initial treatment, while residual psychiatric symptoms were mild, and the patient was fully functional. Therefore, we cannot associate EDB with disease activity or clinical relapse, as this pattern might have persisted throughout this patient’s illness. However, improvement of EDB after second-line treatment with remarkable clinical improvement allows us to postulate that persistent EDB may be associated with ongoing brain dysfunction in this disease and might also be associated with the persistence/recurrence of psychiatric symptoms in this patient.

Other studies documenting electroencephalographic findings following anti-NMDAR encephalitis are required. EEG can identify functional brain abnormalities not reflected in neuroimaging studies. Electroencephalographic patterns such as EDB may serve as markers of residual signs of disease activity, reflecting partially treated or recurrent disease.

## Data Availability

The datasets used and/or analyzed during the current study available from the corresponding author on reasonable request.

## Abbreviations

NMDAR: N-Methyl-D-Aspartate Receptor
EDB: Extreme delta brush
CSF: Cerebrospinal fluid
MRI: Magnetic resonance imaging
EEG: Electroencephalogram
IgG: Immunoglobulin G
CT: Computed tomography
MMSE: Mini–Mental State Examination
IVIG: IntravenousImmunoglobulin
BMI: Body Mass Index
